# *Panax notoginseng* Saponins Protect Cerebral Microvascular Endothelial Cells against Oxygen-Glucose Deprivation/Reperfusion-Induced Barrier Dysfunction via Activation of PI3K/Akt/Nrf2 Antioxidant Signaling Pathway

**DOI:** 10.3390/molecules23112781

**Published:** 2018-10-26

**Authors:** Shaonan Hu, Yali Wu, Bo Zhao, Haiyan Hu, Baochen Zhu, Zongxi Sun, Pengyue Li, Shouying Du

**Affiliations:** School of Chinese Materia Medica, Beijing University of Chinese Medicine, Beijing 100029, China; 20170941185@bucm.edu.cn (S.H.); wuyali1993@163.com (Y.W.); zhaobowua@163.com (B.Z.); yxyhhy@163.com (H.H.); zbcbock123@sina.com (B.Z.); zongxisun@163.com (Z.S.)

**Keywords:** *Panax notoginseng* saponins, oxygen-glucose deprivation/reperfusion, anti-oxidative stress, blood-brain barrier

## Abstract

Oxidative stress plays a critical role in cerebral ischemia/reperfusion (I/R)-induced blood-brain barrier (BBB) disruption. *Panax notoginseng* saponins (PNS) possess efficient antioxidant activity and have been used in the treatment of cerebral ischemic stroke in China. In this study, we determined the protective effects of PNS on BBB integrity and investigated the underlying mechanism in cerebral microvascular endothelial cells (bEnd.3) exposed to oxygen-glucose deprivation/reperfusion (OGD/R). MTT and LDH release assays revealed that PNS mitigated the OGD/R-induced cell injury in a dose-dependent manner. TEER and paracellular permeability assays demonstrated that PNS alleviated the OGD/R-caused disruption of BBB integrity. Fluorescence probe DCFH-DA showed that PNS suppressed ROS generation in OGD/R-treated cells. Immunofluorescence and western blot analysis indicated that PNS inhibited the degradation of tight junction proteins triggered by OGD/R. Moreover, mechanism investigations suggested that PNS increased the phosphorylation of Akt, the activity of nuclear Nrf2, and the expression of downstream antioxidant enzyme HO-1. All the effects of PNS could be reversed by co-treatment with PI3K inhibitor LY294002. Taken together, these observations suggest that PNS may act as an extrinsic regulator that activates Nrf2 antioxidant signaling depending on PI3K/Akt pathway and protects against OGD/R-induced BBB disruption in vitro.

## 1. Introduction

Stroke is one of the leading causes of adult death and long-term disability worldwide. Acute ischemic stroke, resulting from arterial occlusion in the brain, makes up more than 80% of all the cases [1,2]. Nowadays, thrombolytic therapy with recombinant tissue plasminogen activator (rtPA) continues to be an important medical therapy in the management of acute ischemic stroke within 3–4.5 h of symptom onset [3,4]. However, the cerebral ischemia and reperfusion with thrombolysis treatment may result in serious brain injury, such as intracerebral hemorrhagic transformation (HT), with complex pathological mechanisms, and partially due to the oxidative stress and disruption of the BBB [2,4].

The blood-brain barrier (BBB) is a dynamic system that acts as a ‘physical barrier’ to maintain the homeostasis of central nervous system (CNS) by regulating the movement of molecules in and out of the brain [5,6]. Anatomically, BBB is mainly comprised of cerebral microvascular endothelial cells, pericytes, and astrocytic end-feet, together with the noncellular basement membranes (BMs) that surround and separate these cellular constituents from one another [6]. Cerebral microvascular endothelial cells, the core component of BBB, are connected by tight junction proteins (TJs), thus forming the integrated interface with high transendothelial electrical resistance (TEER) and greatly restricting the paracellular diffusion of vascular-derived solutes into the brain [7,8]. The alterations of TJs, particularly claudin-5, occludin and zonula occludens-1 (ZO-1), are associated with BBB dysfunction in many brain diseases, such as acute ischemic stroke [8,9]. During the cerebral ischemia-reperfusion (I/R) period, the overproduction of reactive oxygen species (ROS) is widely regarded as one of the main mechanisms accounting for the direct damage of brain neurons [10]. Meanwhile, excessive ROS may also result in TJs degradation and BBB disruption, which lead exogenous large molecules to freely cross the barrier into the brain and further exacerbate the brain tissue damage indirectly [2,11,12]. However, nowadays, most scientists focus on neurons and brain parenchyma, and direct BBB protection has rarely received research attention [10,11]. Previous research has indicated that early BBB disruption might be a cause rather than a consequence of brain neuron injury [13]. Therefore, the protection of BBB with antioxidants is considered as a potential way to prevent and remedy the I/R injury.

*Panax notoginseng* saponins (PNS) are the main effective constituents of Xuesaitong Injection, which is widely used in the treatment of cerebral ischemic stroke and cardiovascular disease in China [14,15]. PNS have numerous pharmacological effects, including cerebral vasodilation, blood dynamics invigoration, hemostasis, anti-inflammation, anti-apoptosis, anti-thromboembolism, anti-edema, anti-coagulation, anti-hyperglycemia and anti-hyperlipidemia [15,16], and are also reported to protect neurons against OGD/R injury [17]. Moreover, previous studies have shown that PNS and the active ginsenosides have efficient antioxidant activity in vivo and in vitro [18,19,20,21,22,23]. Nuclear factor erythroid 2-related factor 2 (Nrf2), a transcription factor which can regulate endogenous antioxidant defense, plays an active role in the resistance to intracellular ROS [24,25]. It can activate the downstream antioxidant defense enzymes, such as hemeoxygenase 1 (HO-1), to ameliorate the damage from oxidative stress [24,25]. In recent years, Nrf2 has been a promising therapeutic target to prevent oxidative injury in stroke [25]. Furthermore, the activation of Nrf2 after brain injury has been demonstrated to reverse the loss of TJs and prevent BBB disruption, indicating its protective effect on BBB integrity [26,27]. The PI3K/Akt signaling pathway is extensively involved in the regulation of cell proliferation, migration, and survival [28]. Numerous studies have shown that the PI3K/Akt signaling participates in the activation of Nrf2 and regulates the expression of the downstream target proteins [29,30,31,32]. It is reported that PNS protect cardiomyocytes from ischemia-induced apoptosis via activating PI3K/Akt signaling [33], and also have the proangiogenic effects involved with the PI3K/Akt pathway [34]. Meanwhile, total saponins in leaves of *Panax notoginseng* (LPNS) have the antioxidant effect through Nrf2 activation to prevent the primary rat cortical astrocytes and SH-SY5Y neuronal model from the injury of oxidative stress induced by H_2_O_2_ and OGD/R insult, respectively [35]. In addition, PNS inhibit the adhesion events and protect the arteries in atherogenic model relating to the Nrf2 activation [36]. Moreover, panaxatriol saponins in *Panax notoginseng* activate endogenous cytoprotective mechanism through PI3K/Akt/Nrf2/HO-1 signaling pathway and attenuate OGD/R-induced oxidative injury in PC12 cells [37]. However, the protective effects of PNS on cerebral microvascular endothelial cells, especially their barrier function, with OGD/R insult and the underlying mechanisms have not been investigated yet.

In the current research, we demonstrated that PNS could ameliorate the OGD/R-induced injury of bEnd.3 cells and disruption of BBB barrier function, which were related to the suppression of ROS generation and TJs degradation. In addition, we revealed that these effects might be mediated by Nrf2 activation depending on the PI3K/Akt signaling pathway. This work provides strong evidence that PNS may benefit acute ischemic stroke with thrombolytic treatment by the means of BBB protection through anti-oxidative stress.

## 2. Results

### 2.1. PNS Alleviated the Injury of bEnd.3 Cells after OGD/R Insult

**Figure 1:**
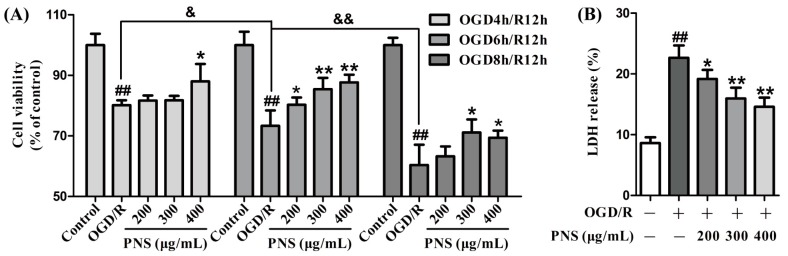
The protective effects of PNS against OGD/R-induced injury of bEnd.3 cells. (**A**) MTT assay to evaluate the protective effects of PNS against OGD/R-induced decrease of cell viability in different injury time. (**B**) LDH release assay to further confirm the protective role of PNS on OGD6h/R12h-induced cytotoxicity. Data are expressed as mean ± SD (N = 4). ^&^ *p* < 0.05, ^&&^ *p* < 0.01, ^##^ *p* < 0.01 vs. Control group, * *p* < 0.05 and ** *p* < 0.01 vs. OGD/R group.

To ascertain the protective effects of PNS against OGD/R-induced injury, we first conducted a 3-(4,5-dimethylthiazol-2-yl)-2,5-diphenyltetrazolium bromide (MTT) assay to examine the cell viability in different treatment groups. As displayed in Figure 1A, the viability of bEnd.3 cells was significantly decreased in different OGD/R groups compared to the control group. Noticeably, the decrease was more distinct with the extension of injury time (decreased from 19.9% in OGD4h/R12h to 39.7% in OGD8h/R12h). As a whole, different concentrations of PNS (200 μg/mL to 400 μg/mL) could increase the cell viability more obviously in OGD6h/R12h group (up to 19.6%) compared with the other two groups (Figure 1A). To further confirm the protective role of PNS, we next evaluated the cytotoxicity in bEnd.3 cells following OGD6h/R12h insult by LDH release assay. As shown in Figure 1B, cytotoxicity in OGD/R group increased by approximately 2.5 times compared to the normal control group, and PNS intervention reduced the cytotoxicity by up to 35.5%. Taken together, these results suggested that PNS alleviated OGD/R-induced injury of bEnd.3 cells.

### 2.2. PNS Attenuated the OGD/R-Induced Disruption of In Vitro BBB Integrity

**Figure 2:**
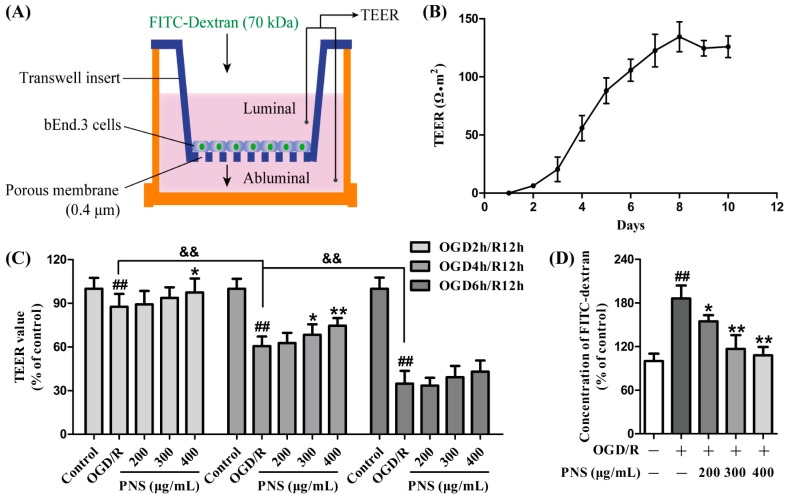
The protective effects of PNS against OGD/R-induced disruption of in vitro BBB integrity. (**A**) Illustration of the in vitro BBB model that bEnd.3 cells were seeded on a membrane of transwell insert, and the TEER and paracellular diffusion of FITC-dextran (70 kDa) were measured. (**B**) The barrier function of the bEnd.3 cell monolayer was evaluated by TEER value for 10 days. (**C**) The TEER assay to evaluate the protective effect of PNS against OGD/R-induced disruption of barrier function in different injury time. (**D**) The paracellular diffusion assay to further confirm the protective role of PNS on OGD4h/R12h-induced barrier disruption. Data are expressed as mean ± SD (N = 3). ^&&^ *p* < 0.01, ^##^ *p* < 0.01 vs. Control group, * *p* < 0.05 and ** *p* < 0.01 vs. OGD/R group.

To evaluate the effects of PNS on the in vitro BBB integrity under OGD/R condition, bEnd.3 cells were cultured in transwell inserts under normoxia for 8 days until the peak TEER value (Figure 2A,B). Further investigation suggested that the TEER value of the bEnd.3 monolayer decreased in a similar manner with the cell viability under different OGD/R conditions (decreased from 12.4% in OGD2h/R12h to 65.3% in OGD6h/R12h) (Figure 2C). However, inconsistent with the results of MTT assay, PNS showed no noticeable protective effect against the TEER decrease in OGD6h/R12h group, but distinct recovery in OGD4h/R12h group (up to 23.2%). Furthermore, PNS also showed a weak protective effect against TEER decrease in OGD2h/R12h group. The barrier protective effect of PNS was further demonstrated by the paracellular diffusion of FITC-dextran (70 kDa) across the cell monolayer subjected to OGD4h/R12h. As shown in Figure 2D, the concentration of FITC-dextran in the lower chamber (abluminal) was significantly increased in OGD/R group (86.1% versus control group), and noticeably decreased with PNS intervention (up to 42.1% versus OGD/R group). These date suggested that PNS treatment could improve microvascular endothelial barrier function insulted by moderate OGD/R injury.

### 2.3. PNS Inhibited the OGD/R-Induced ROS Generation in bEnd.3 Cells

**Figure 3:**
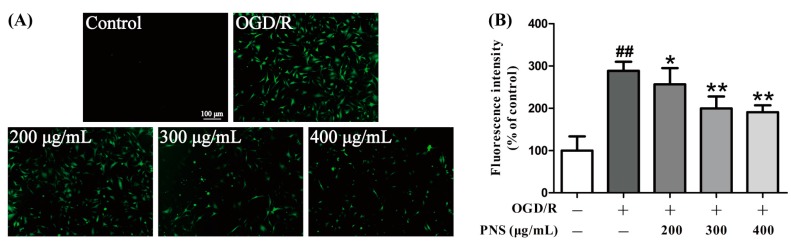
The inhibitory effect of PNS on the OGD4h/R12h-induced ROS production. DCFH-DA, a fluorescent indicator, was used to visualize ROS, which was detected by fluorescence microscopy (**A**) and quantified by microplate reader (**B**). Data are expressed as mean ± SD (N = 6). ^##^ *p* < 0.01 vs. Control group, * *p* < 0.05 and ** *p* < 0.01 vs. Model group. Scale bar = 100 μm.

The intracellular ROS level was detected by DCFH-DA, a fluorescent reagent that acts as ROS indicator. The results of fluorescence detection demonstrated that ROS level was significantly increased in OGD4h/R12h group compared to the control group. This phenomenon is partially blocked by the PNS treatment. Concentrations of 300 μg/mL and 400 μg/mL notably decreased the intracellular ROS level, compared to the OGD/R group (Figure 3A).

We also quantified the fluorescence intensity using a microplate reader. The graph showed a similar result as fluorescence microscopy (Figure 3B). These findings revealed that PNS inhibited the OGD/R-induced ROS production in bEnd.3 cells.

### 2.4. PNS Mitigated the OGD/R-Induced Degradation of Tight Junction Proteins

**Figure 4:**
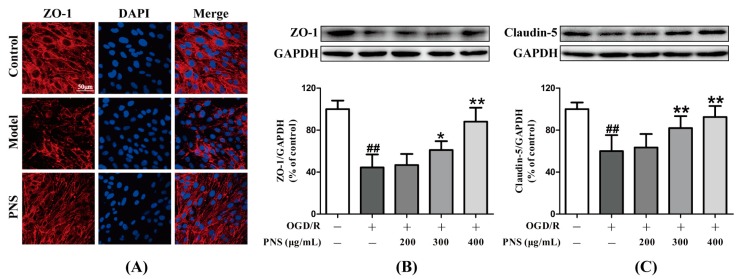
The protective effects of PNS against OGD4h/R12h-induced degradation of TJ proteins. (**A**) Representative confocal microscopy images showing the immunostaining of ZO-1 after OGD/R and PNS (400 μg/mL) intervention in bEnd.3 cells. Scale bar = 50 μm. The expression levels of ZO-1 (**B**) and claudin-5 (**C**) were measured by Western blot. The band intensities were assessed by Image J software and normalized against the GAPDH signal. Data are expressed as the mean ± SD (N = 3). ^##^ *p* < 0.01 vs. Control group, * *p* < 0.05 and ** *p* < 0.01 vs. Model group.

To further demonstrate the protective effects of PNS on the integrity of the barrier with OGD/R challenge, we investigated the expression of ZO-1, a tight junction protein, by immunocytochemistry (Figure 4A). In addition, the expression of ZO-1 and claudin-5 were evaluated by western blot analysis (Figure 4B,C). OGD4h/R12h injury obviously decreased the tight junction expression compared to the control group. PNS treatment, especially with the concentration of 400 μg/mL, could upregulate the decreased expression of ZO-1 and claudin-5 after OGD4h/R12h. These results indicated that PNS mitigated the degradation of tight junction proteins induced by OGD/R insult, which might further maintained the integrity of the bEnd.3 monolayer.

### 2.5. PNS Activated Akt-Nrf2 Relating Pathway in bEnd.3 Cells Subjected to OGD/R

**Figure 5:**
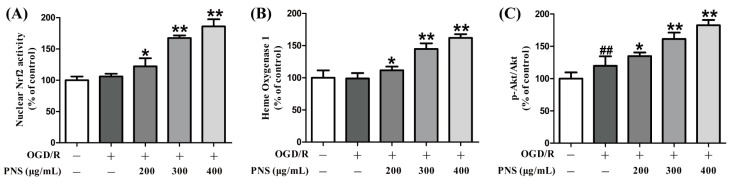
Effects of PNS on the upregulation of the expression of Akt-Nrf2 pathway relative proteins in bEnd.3 cells after OGD4h/R12h challenge. (**A**) A transcription factor assay kit was used to determine the activity of nuclear Nrf2 which binds to a specific dsDNA sequence containing the Nrf2 response antioxidant response element in the wells. ELISA assays to determine the HO-1 expression (**B**) and p-Akt/Akt ratio (**C**) in bEnd.3 cells after OGD/R and PNS treatment. Data are expressed as mean ± SD (N = 6). ^##^ *p* < 0.01 vs. Control, * *p* < 0.05 and ** *p* < 0.01 vs. Model.

To illuminate the underlying mechanism of the protective effects of PNS, we utilized commercial kits and performed ELISA assays (Figure 5). Nrf2 is a key transcription factor that triggers the expression of antioxidant proteins, including HO-1, under specific conditions. The results of our studies revealed that nuclear Nrf2 activity, together with its downstream protein HO-1 expression, were not altered significantly after exposure to OGD4h/R12h. However, both of them were significantly upregulated with the treatment of PNS compared with the OGD/R group (Figure 5A,B). Next, according to the previous studies [30,31,32], we hypothesized the PI3K/Akt as the upstream signal to modulate the Nrf2/HO-1 pathway in our investigation. The quantitative graph in Figure 5C showed that the p-Akt/Akt ratio was upregulated in OGD/R group compared with the level in the control group. Moreover, the ratio was further increased with the intervention of PNS despite the OGD4h/R12h insult (Figure 5C).

**Figure 6:**
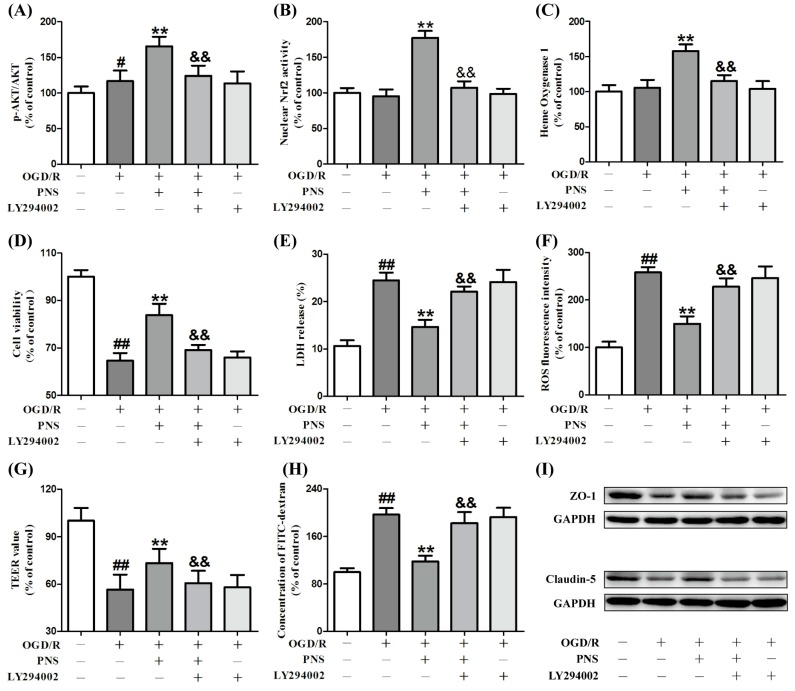
Effects of PNS on bEnd.3 cells with OGD/R injury were inhibited by LY294002. Upregulation of p-Akt/Akt ratio (**A**), nuclear Nrf2 activity (**B**) and HO-1 expression (**C**) were almost inhibited by LY294002. The protective effects of PNS on cell viability (**D**) and cytotoxicity (**E**) were almost inhibited by LY294002. (**F**) The inhibitory effect of PNS on ROS production was almost inhibited by LY294002. The protective effects of PNS on barrier integrity (**G**,**H**) and tight junction disruption (**I**) were almost inhibited by LY294002. LY294002 had no significant effect on OGD/R- treated cells compared to the OGD/R group. Data are expressed as mean ± SD (N > 3). ^##^ *p* < 0.01 vs. Control, ** *p* < 0.01 vs. Model, ^&&^ *p* < 0.01 vs. PNS treatment group.

Interestingly, LY294002, a specific inhibitor of PI3K, mostly inhibited the effects of PNS on the upregulation of p-Akt/Akt ratio, nuclear Nrf2 activity and HO-1 expression (Figure 6A–C). 

Furthermore, all the protective effects described above, such as cell viability, cytotoxicity, ROS level, monolayer integrity or TJs expression, were diminished with the interference of LY294002 (Figure 6D–I). These date revealed that PNS could activate Nrf2 in bEnd.3 cells subjected to OGD/R and that these effects were PI3K/Akt dependent.

## 3. Discussion

Ischemic stroke is a leading cause of adult death and disability worldwide, with oxidative stress playing a crucial role in the injury mechanism of thrombolytic therapy [28,38]. Accumulating evidence indicates that oxidative stress injures endothelial cells, degrades TJs and contributes to an increase in BBB permeability [26,39,40]. It has been demonstrated that BBB disruption might be the a cause rather than a consequence of brain neuron injury and increase the risk of intracerebral hemorrhagic transformation in ischemic stroke [4,13]. However, scientists almost focus their attention on neurons and brain parenchyma during the stroke treatment and overlook the direct BBB protection [10,11]. PNS are widely used in the prevention and treatment of cerebral ischemic stroke in China and reported to protect neurons against OGD/R insult [14,17,35]. Previous studies have proved that PNS or the active compounds could reduce ROS generation, alleviate oxidative stress-induced injury [20,21,23], and also have the ability to activate Nrf2 under different pathological conditions [35,36,37]. However, to our knowledge, it has not been illustrated whether PNS have the protective effects against I/R-induced injury of brain microvascular endothelial cell and disruption of BBB integrity. In addition, the underlying mechanisms have not been investigated yet. In our study, ROS level was significantly elevated in bEnd.3 cells with the stimulation of OGD/R, which resulted in the decreased cell viability and increased cytotoxicity. Meanwhile, excessive ROS also disrupted the BBB integrity, which is featured by the decreased TEER, increased permeability and reduced TJs expression. Conversely, PNS treatment suppressed ROS generation and alleviated the injuries above, especially sustained the integrity of in vitro BBB model. A key finding of this study was that Nrf2 might play a pivotal role in the protective effects of PNS, and the impact of PI3K inhibitor revealed that the upregulation of nuclear Nrf2 activity might be mediated through PI3K/Akt signaling pathway. All these results indicated that the protective effects of PNS on BBB endothelial cells were closely related to the antioxidant activity.

To embody the protective effects of PNS in the greatest extent, the appropriate injury time (degree) of OGD/R was firstly explored in MTT assay, covering from OGD2h/R12h to OGD10h/R12h with the interval of 2 h (data not fully shown in Figure 1A). It was assumed that moderate injury (OGD4h/R12h) might influence the cell proliferation with little cell death, and the decreased cell viability in OGD/R group was due to the decreased cell proliferation compared to the control group. Actually, it was demonstrated that the cell viability was not significantly influenced by OGD4h/R12h insult when the cells were completely confluent, but decreased by approximately 8.2% with OGD6h/R12h injury (data not shown). In our study, PNS afforded the best protective effects in OGD6h/R12h group, while no noticeable effects in OGD2h/R12h or OGD10h/R12h. This phenomenon might be explained by the injury degree. Too severe injury (cell viability decreased by 53.2% in OGD10h/R12h) might lead to serious influence of cell proliferation accompanied with large-scale cell death that PNS could not alleviate. While too mild injury (cell viability decreased by 1.8% in OGD2h/R12h) might just result in weak influence of cell proliferation, which compressed the protective effect of PNS. Further, the protective effect of PNS on OGD6h/R12h injury was confirmed via LDH release assay. In the research of the in vitro BBB integrity in TEER assay, it was found that the injury of OGD6h/R12h is not appropriate that PNS lost the protective role. Then the injury time was shortened to OGD4h/R12h and the protective effects of PNS re-emerged. This difference might also result from the influence of injury degree. Compared to the OGD4h/R12h group, OGD6h/R12h resulted in more TJs loss accompanied with cell death, and led to severer damage of BBB integrity that PNS could not mitigate. In another word, just like the OGD10h/R12h-induced serious injury of cell viability in MTT assay, OGD6h/R12h resulted in relatively severe damage of BBB integrity, and this degree of damage was not appropriate for the evaluation of the PNS protection against the barrier disruption. As a result, OGD4h/R12h was considered as the appropriate injury time in the following experiments.

Excessive reactive oxygen species (ROS) are produced during brain ischemia/reperfusion and contribute to a series of injury including BBB disruption, inflammation and cell apoptosis [25]. Previous investigations have indicated that antioxidants could inhibit ROS generation and attenuate BBB disruption after cerebral ischemia/reperfusion [2,11]. In the present study, intracellular ROS were significantly upregulated with the OGD/R stimulation, along with the disruption of the bEnd.3 monolayer integrity, as evidenced by the decreased TEER and increased FITC-dextran extravasation. PNS efficiently suppressed the ROS generation due to the antioxidant activity and mitigated the damage of the in vitro BBB integrity. During the period of FITC-dextran diffusion, the transwell inserts were kept under a consistent 37 °C condition, which could not be achieved during the TEER measurement. The slight distinction of the experimental temperature accompanied with different cell state might lead to better protective effect of PNS in the paracellular permeability assay (Figure 2C, middle group) than the TEER experiment (Figure 2D). ZO-1 is a major protein in tight junctions, which are crucial to maintain the BBB integrity [41]. Claudin-5 is reported to be an essential molecule that aggravates the disruption of BBB in I/R condition [2]. Both of them are susceptible to ROS attack [42]. To further demonstrate the protective effects of PNS on impaired BBB function, the expression of ZO-1 and claudin-5 was examined in bEnd.3 cells with OGD/R insult. The results suggested that PNS might prevent BBB disruption during I/R injury by inhibiting degradation of ZO-1 and claudin-5 tight junction proteins through antioxidation.

In recent years, researchers have paid particular attention to the PI3K/Akt/Nrf2/HO-1 pathway that plays a pivotal role in the resistance to oxidative stress [43,44,45]. Nrf2 is a transcription factor that can regulate endogenous antioxidant defense and also a molecular target for pharmacological resistance to oxidative damage [24,25]. Actually, under physiological conditions, Nrf2 is initially located in cytoplasm inactively due to its combination with Keap1 [29]. Once stimulated by external stimuli, Nrf2 dissociates from the complex and subsequently translocates into the nucleus, where it binds to the antioxidant response element (ARE) with musculoaponeurotic fibrosarcoma (Maf) protein and activates the expression of related phase II detoxifying antioxidant enzymes [29,46]. HO-1 is a critical downstream antioxidant enzymes of Nrf2. It has been suggested to be effective in protecting against OGD/R-induced neuronal injury and promoting antioxidant protection [46]. In addition, Nrf2 activator has been demonstrated to mitigate hemorrhagic transformation in cerebral ischemia by upregulating the TJs expression and maintaining the BBB integrity [47,48]. In our study, the DNA-binding activity of Nrf2 that translocated into the nucleus was significantly upregulated in the PNS treatment groups compared with OGD/R group, and the expression of downstream HO-1 was also upregulated with PNS intervention. Whereas, neither of them was significantly influenced by OGD/R challenge, which was consistent with the preceding study [12]. Previous reports show that the activation of PI3K/Akt signaling can trigger the nuclear translocation of Nrf2 to suppress ROS and promote cell survival [43,45]. Actually, Nrf2 activation can also be regulated through other pathways, such as MAPK kinases (JNK, ERK, and p38), but panaxatriol saponins in *Panax notoginseng* have no effect on the phosphorylation of these MAPK kinases [37]. Thus, the potential effect of PNS on the activation of PI3K/Akt was hypothesized based on the previous reports [33,34]. In this study, the results showed that OGD/R slightly upregulated Akt phosphorylation, and PNS treatment further stimulated the phosphorylation of Akt despite the influence of OGD/R. Interestingly, the activation of Nrf2, inhibition of ROS and protection of BBB integrity were reversed when the expression of p-Akt was down-regulated by LY294002, a PI3K inhibitor that disturbed PI3K/Akt pathway. Taken together, these results suggested that the effects of PNS are related to the activation of Nrf2/HO-1 pathway via PI3K/Akt signaling.

Several ginsenosides in PNS are potential phytoestrogens, and structurally and functionally similar to 17β-estradiol [49]. Previous studies have demonstrated that estrogen receptor is related to the activation of PI3K/Akt signaling by Ginsenoside Rg_1_, and PI3K/Akt/Nrf2 by Notoginsenoside R_1_ or Ginsenoside Rb_1_ [50,51,52]. Thus, we speculated that the estrogen receptor might be involved in the effects of PNS illustrated above, and further investigations need to be carried out. PI3K/Akt pathway is reported to play an important role in the promotion of cell proliferation and survival [53], and the regulation of claudin-5 expression [54]. Whether PI3K/Akt is directly involved in the PNS protection of the cell viability and the BBB integrity remains to be studied. What’s more, which main components play a major role in the protective effects, and whether different active constituents combinations exist synergistic effects should also be further examined. In addition, the limitation of this research is that whether the signaling pathway can be regulated by PNS under normal culture condition was not confirmed. Moreover, further research direction can also be transferred to determine the protective effects of PNS relating to inflammation, which are resulted from excessive ROS generation as described above. In a word, these in vitro studies supported our hypothesis that PNS protected against OGD/R-induced cerebral microvascular endothelial cell injury and BBB disruption via the activation of Nrf2/HO-1 antioxidant pathway depending on PI3K/Akt signaling.

## 4. Materials and Methods

### 4.1. Materials

**Figure 7:**
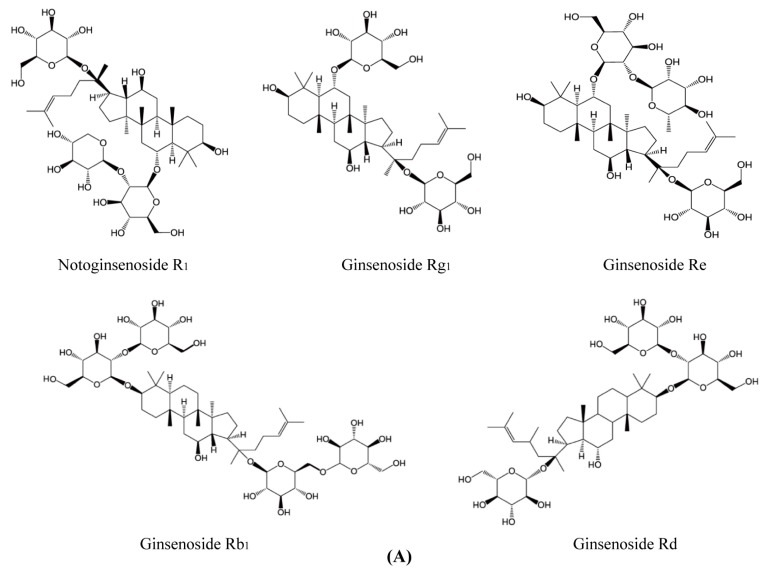

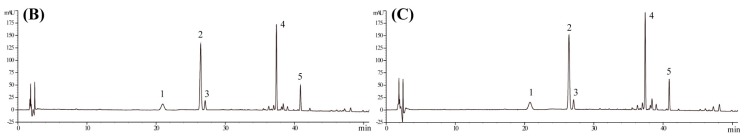
(**A**) Chemical structures of the main compounds in *Panax notoginseng* saponins. (**B**) Chromatogram profiles of PNS reference. (**C**) Chromatogram profiles of PNS extract. The main components of PNS in the chromatograms are as following: 1. Notoginsenoside R_1_; 2. Ginsenoside Rg_1_; 3. Ginsenoside Re; 4. Ginsenoside Rb_1_; 5. Ginsenoside Rd. UV absorbance of the HPLC samples was monitored at 203 nm.

PNS extracted from *Panax notoginseng* were obtained from Yunnan Sanqi Technology Co. Ltd. (Wenshan, China). As determined by HPLC (Figure 7B,C), the contents of each ingredient in PNS extract were notoginsenoside R_1_ (9.05%), ginsenoside Rg_1_ (30.02%), ginsenoside Re (3.81%), ginsenoside Rb_1_ (31.58%), ginsenoside Rd (9.40%) (Figure 7A) and met the Chinese Pharmacopoeia (2015 edition) criterion. PNS reference was purchased from National Institutes for Food and Drug Control (Beijing, China). High-glucose Dulbecco’s modified Eagle’s medium (DMEM), fetal bovine serum (FBS), penicillin and streptomycin were purchased from Gibco (Thermo Fisher Scientific Co., Waltham, MA, USA). The primary antibodies against ZO-1, claudin-5, and GAPDH were purchased from Abcam (Cambridge, MA, USA).

### 4.2. Cell Culture

Mouse microvascular cerebral endothelial cells (bEnd.3) (CRL-2299, ATCC, Manassas, VA, USA) were cultured in DMEM supplemented with 10% FBS, 100 U/mL penicillin and 100 U/mL streptomycin (normal culture medium) at 37 °C under a humidified atmosphere containing 5% CO_2_. Cells were passaged every 3–4 days until grown to 80–90% confluence. Culture medium was replaced after 24 h of passaging and every 2 days thereafter. Experiments were performed with cells from passages 5 to 25.

### 4.3. Oxygen-Glucose Deprivation/Reperfusion (OGD/R) Insult

To mimic ischemia/reperfusion-like conditions in vitro, we exposed bEnd.3 cells to oxygen-glucose deprivation followed by reperfusion as previously described with modification [11]. In brief, bEnd.3 cells with appropriate confluence were washed three times with PBS, and the normal culture medium was replaced with DMEM medium without glucose. Then the cells were transferred to an anaerobic chamber under 95%N_2_/5%CO_2_ (MIC-101; Billups-Rothenberg, Del Mar, CA, USA) and incubated at 37 °C for a specified period of time. Following oxygen-glucose deprivation insult, cells were incubated for another 12 h under normal culture condition.

### 4.4. Drug Treatment

The present study was divided into five groups: (1) Control group, bEnd.3 cells were cultured under normal culture condition for the same period of time with OGD/R insult; (2) Model group (OGD/R group), bEnd.3 cells were insulted by oxygen-glucose deprivation/reperfusion; (3) Low PNS group, bEnd.3 cells were treated with 200 μg/mL PNS for 12 h before OGD; then treated with 200 μg/mL PNS for another 12 h in reperfusion period; (4) Middle PNS group (300 μg/mL PNS); (5) High PNS group (400 μg/mL PNS). 

**Figure 8:**
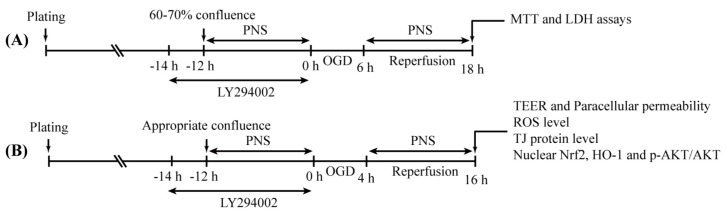
Experimental timeline for different experiments. (**A**) Timeline for MTT and LDH release assays. (**B**) Timeline for the measurement of TEER and paracellular permeability (peak TEER); ROS level (60–70% confluence); TJ protein level, nuclear Nrf2 activity, HO-1 level and p-Akt/Akt ratio (90% confluence).

For PI3K/Akt inhibition, the PI3K inhibitor LY294002 (Selleckchem, Burlington, NC, USA) was added to the culture medium 2 h before PNS treatment and together with PNS before OGD/R. Experimental timelines of PNS with/without LY294002 treatment in bEnd.3 cells exposed to OGD/R in different experiments were summarized in Figure 8.

### 4.5. Cell Viability Measurement

Cell viability was measured through MTT assay. In brief, bEnd.3 cells were cultured in 96-well plates and subjected to OGD/R insult or drug treatment as described above. Then, 20 μL of MTT (5 mg/mL; KeyGen Biotech, Nanjing, China) was added and continuously incubated at 37 °C for another 4 h in the dark. The medium was replaced with 150 μL of DMSO to solubilize the purple formazan product with shaking for 15 min. The OD values were measured with a Multiskan Go microplate reader (Thermo, Waltham, MA, USA) at a detection wavelength of 490 nm. Cell viability was expressed as a percentage of the control group.

### 4.6. Cell Cytotoxicity Measurement

Cell cytotoxicity was evaluated through the LDH release assay. Briefly, bEnd.3 cells were cultured in 96-well plates and subjected to OGD/R insult or drug treatment as described above. After treatment, the LDH released from cells was detected using a commercial LDH assay kit (KeyGen Biotech, Nanjing, China) according to the manufacturer’s instructions. Absorbance was measured at 490 nm and the LDH release rate was calculated as the followed formula: LDH release rate (%) = [OD (treatment) − OD (blank)]/[OD (maximum) − OD (blank)] × 100%.

### 4.7. Trans-Endothelial Electrical Resistance (TEER) Assay

To evaluate the integrity of the bEnd.3 cell monolayer, the TEER value was measured with a Millipore Millicell ERS system equipped with chopstick electrodes (Millipore, Billerica, MA, USA) as previously described with modification [27]. Briefly, the cells were seeded into the upper ‘apical’ chamber of transwell inserts (3460; Corning, NY, USA) with the density of 2 × 10^5^ cells/well in 0.5 mL DMEM normal culture medium, while 1.5 mL of the same medium was added to the lower chamber to prevent the formation of an oncotic pressure gradient. The TEER values were measured every day with regular intervals until the bEnd.3 cells reached a peak resistance. The values were standardized by the area of the culture inserts and shown as Ω·cm^2^ by subtracting the resistance of blank filters (i.e., no cells) from that of the sample resistance.

### 4.8. Paracellular Permeability Measurement

To evaluate the paracellular permeability, diffusion of FITC-dextran (70 kDa; Sigma-Aldrich, St. Louis, MO, USA) across the integrated monolayer was measured as previously described with modification [55]. In brief, following OGD/R injury with/without drug treatment, the inserts were transferred into fresh wells containing pre-warmed HBSS buffer (KeyGen Biotech, Nanjing, China) and washed twice with the buffer. Then FITC-dextran (1 mg/mL) was added to the upper chamber and incubated at 37 °C for 60 min in the dark. Inserts were then removed from the wells and the sample solutions were collected from the lower chamber and transferred into black 96-well plates (3614; Corning, NY, USA). Then, the fluorescence intensity was measured using fluorescence microplate with an excitation and emission wavelength of 485 and 525 nm, respectively. The concentrations of FITC-dextran in the lower chamber were calculated by the calibration curve of the fluorescence units of known FITC-Dextran concentrations and expressed as the percentage of the corresponding normoxic cells.

### 4.9. Determination of Intracellular ROS

The intracellular ROS level in each group was measured by the fluorescent change resulting from oxidation of DCFH-DA (KeyGen Biotech), a fluorescent probe with membrane permeability [56]. After the OGD/R injury or drug treatment, cells were washed with PBS and treated with 10 μM DCFH-DA at 37 °C for 30 min in dark. Then the cells were washed three times with serum-free DMEM to remove the free molecules of the probe. The fluorescent intensity of intracellular ROS was detected by fluorescence microplate (SpectraMax i3x; Molecular Devices, San Jose, CA, USA) and epi-fluorescence microscopy (IX-71; Olympus, Tokyo, Japan). To increase the precision for ROS measurement with fluorescence microplate, DMSO/PBS (90%/10%) was added for cell lysis after the last DMEM wash [57].

### 4.10. Immunofluorescence Assay

The bEnd.3 cells were cultured in Laser confocal Petri dish until 90% confluence. After the corresponding treatment, the cells were fixed with 4% paraformaldehyde, permeabilized with 1% Triton X-100 and incubated with primary antibody against ZO-1 (1:50), followed by incubation with the secondary antibody of TRITC-conjugated Goat Anti-Rabbit IgG (1:100; ZSGB-BIO, Beijing, China) and 4′,6-Diamidino-2-phenylindole (DAPI, 1:100; Solarbio, Beijing, China). Immunostaining was examined by a confocal microscope (Olympus).

### 4.11. Western Blot Analysis

After each treatment, the cell pellets were lysed in ice-cold RIPA buffer (Solarbio) containing PMSF. The cell lysates were centrifuged at 12,000 rpm at 4 °C to produce the whole cell extracts, and the protein concentration was quantified using the BCA protein assay kit (Solarbio). Sample loading buffer was added and boiled at 95 °C. Equal amounts of protein were separated on a 10% SDS-PAGE and subsequently transferred to PVDF membranes (Millipore). After blocking with 5% non-fat milk, the membranes were incubated at 4 °C overnight with primary antibodies against ZO-1 (1:300), claudin-5 (1:300) or GAPDH (1:5000), and further incubated with horseradish peroxidase-conjugated secondary antibodies. The protein bands were washed and developed with enhanced chemiluminescence reagents (ECL, Vazyme Biotech, Nanjing, China) and scanned with the Kodak Digital Imaging System. The optical density (OD) values of each band were normalized to GAPDH using Image J software (version 1.37, NIH, Bethesda, MD, USA).

### 4.12. Detection of Nuclear Nrf2 Activity

Nuclear extracts were used to quantify the Nrf2 DNA-binding activity by Nrf2 Transcription Factor Assay Kit (Abcam 207223) using a colorimetric technique according to the manufacturer’s instructions. This kit was designed to rapidly quantify the activated Nrf2, which binds to a specific double stranded DNA (dsDNA) containing the Nrf2 response antioxidant response element (ARE) and immobilized to each well of 96-well plate.

### 4.13. Enzyme-linked Immunosorbent Assay (ELISA) to p-Akt, Akt, and HO-1

Quantification of heme oxygenase 1 (HO-1) was carried out by Heme Oxygenase-1 (HO-1) Mouse SimpleStep ELISA kit (Abcam 204524) according to the manufacturer’s instructions. Determination of the p-Akt/Akt ratio was carried out by Akt (pS473) + total Akt ELISA Kit (Abcam 126433) according to the manufacturer’s instructions.

### 4.14. Statistical Analysis

Statistical analysis was undertaken by one-way analysis of variance (ANOVA) with SPSS 20.0 (IBM, Armonk, NY, USA). All results were expressed as mean ± SD from at least three independent measurements and statistical significance was set at *p* < 0.05.

## 5. Conclusions

**Figure 9:**
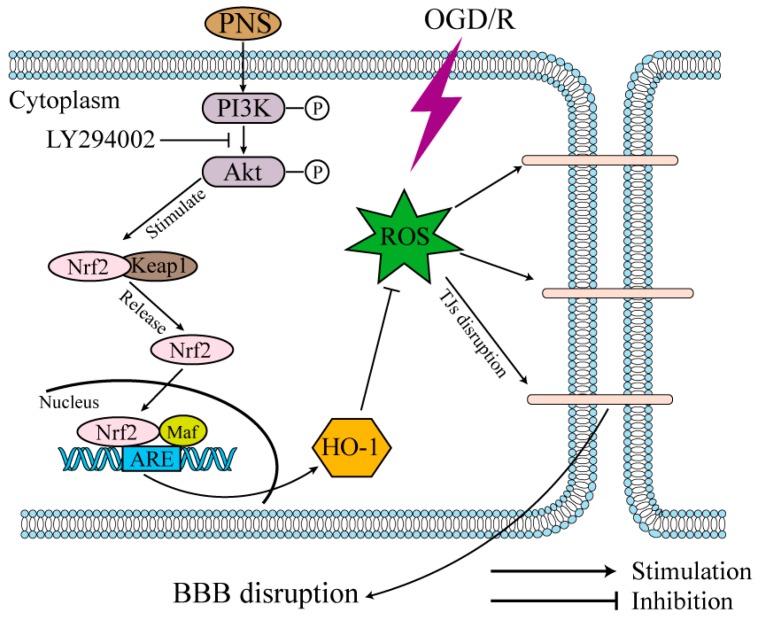
A proposed schematic diagram of the PNS protection on BBB endothelial cells. OGD/R induces intracellular ROS accumulation and causes oxidative stress, which leads to endothelial cell injury and BBB disruption. PNS stimulated PI3K/Akt signaling, which causes Nrf2 nuclear translocation and activation, and further enhance the downstream HO-1 expression. Finally, HO-1 prevents endothelial cells from oxidative stress-induced cell injury and BBB disruption.

The present study provided that *Panax notoginseng* saponins could protect against oxygen-glucose deprivation/reoxygenation-induced injury of cerebral microvascular endothelial cell and disruption of in vitro BBB integrity. The underlying mechanism was relating to the anti-oxidative stress through activation of Nrf2 antioxidant signaling depending on the PI3K/Akt pathway (Figure 9). This study might develop a new application of PNS as an adjuvant therapy for acute ischemic stroke in thrombolytic treatment, to maintain the BBB integrity and further reduce the risk of intracerebral hemorrhagic transformation and brain neuron injury. In addition, PNS have the pharmacological effects of cerebral vasodilation and blood dynamics invigoration, which may further exert the efficacy enhancing and toxicity reducing in the adjuvant therapy of acute ischemic stroke.
